# *Response to Prof D. Vanden Berghe letter:* 'There are not enough data to conclude that Monomethylsilanetriol is safe’

**DOI:** 10.1186/1743-7075-10-65

**Published:** 2013-10-22

**Authors:** Ravin Jugdaohsingh, Simon HC Anderson, Stephen D Kinrade, Jonathan J Powell

**Affiliations:** 1 MRC Human Nutrition Research, Elsie Widdowson Laboratory, Fulbourn Road, Cambridge CB1 9NL, UK; 2 Gastrointestinal Laboratory, Rayne Institute, St Thomas’ Hospital, London, UK; 3 Department of Chemistry, Lakehead University, Thunder Bay, ON, Canada

**Keywords:** Monomethylsilanetriol, Choline-stabilised orthosilicic acid, Nanoparticles, Orthosilicic acid, Proton nuclear magnetic resonance spectroscopy

## Background & discussion

The silicon supplement “ch-OSA [choline stabilized 'orthosilicic’ acid] was developed by Dirk Vanden Berghe for Bio Minerals n.v.” [1-4]. Here, Dirk Vanden Berghe provides a list of comments/questions on our recent publication [5] concerning a different form of supplemental silicon, namely 'Monomethylsilanetriol’ (MMST; Si(OH)_3_CH_3_).

We entirely disagree with his concerns but before addressing these directly, we wish to re-emphasise important background information. Germane to this is the understanding of the limitations and concerns over current supplemental forms of silicon, as dictated by their aqueous chemistry.

From the diet silicon is absorbed as Si(OH)_4_ (orthosilicic acid) [6-8]. Any effort to concentrate this form of silicon leads to self-association, polymerisation and (nano) silica formation. In ch-OSA, for example, a high dose quaternary ammonium compound stabilises these very small nanoparticles of silica to prevent their aggregation [9] as, for example, is discussed by Iler [10]. However, in the case of silicon dioxide/silicic acid, no stabilisers are used and aggregation may proceed with mixed soluble, nano and micron sized silica species (note, in this case, silicic acid is used as a 'catch-all’ for monomeric and varying degrees of polymeric silica). For all of these supplemental forms, bioavailability is limited (absorption less than 20% and as low as 1%) [8]. Moreover, there are currently significant concerns over nanosilica and more careful evaluation is required for its safe ingestion [11,12]. We thus reported on the metabolism and human safety of a *soluble* silicon form (MMST) that is very well absorbed (i.e. equivalent to dietary orthosilicic acid [8,13]).

### Direct responses

Overall, our intention was not to provide a full toxicological report of MMST but, rather, and as stated in our paper [5], to establish if it contributes to the body pool of silicon in humans and, if so, whether it was safe in that setting. We stand firmly by our conclusions that, in both cases, the answers are “yes”: MMST provided a marked increase in the body pool of Si according to state-of-art measures and was safe throughout the study. Nonetheless, we fully agree that for a formal safety assessment of MMST, or any (silicon) supplement, human safety data should be accompanied by specific, preclinical toxicological data and, as Professor Vanden Berghe notes, be judged in Europe by the EFSA rather than by individual academics. Our following responses deal with the points of his main letter in chronological order:

1. We do not agree that EFSA previously gave a “negative” opinion on the use of MMST as a supplement. They were unable to reach a conclusion as insufficient data were provided [14]. The assessment process appears, nonetheless, ongoing, underpinning the importance of new findings such as those provided in our manuscript [5].

2. In the introduction of our paper, previous studies, when taken together, help to provide a rationale for our study: they add weight to arguments over the human safety of MMST rather than being conclusive in their own right. Indeed, this is partly why we undertook a more direct human safety study. As stated in the introduction of our paper, MMST is used widely in continental Europe without any reported adverse effects.

3. We concur with EFSA guidance that long term human safety studies are *not* required for safety assessment of food supplements. However, as noted above, at least some appropriate pre-clinical toxicological data *should* be provided. In the context of diet and dietary supplement assessment, the study of 22 human subjects over 4 weeks provides significant and relevant added value to toxicological assessment in rodents. Indeed, again using ch-OSA as an example of one of the approved European silicon supplements, we note the EFSA’s actual comments of Section 3.3 in their report [15]; “*The petitioner states that no complete toxicological evaluation was carried out for ch-OSA since it contains choline and a stabilised form of a natural occurring silicon compound (orthosilicic acid). Instead, the petitioner provided the results of a large number of animal and human supplementation studies”.*

Notwithstanding that orthosilicic acid should read nanosilica, it makes the valid and important point that dietary supplements do *not* carry the same onus of toxicological assessment as therapeutics. Data may, more commonly, be bridged and evidence is usually drawn from both direct rodent studies and human supplementation studies. The process is robust but, equally, recognises the distinction between a “food” and a “therapeutic”.

4. We do not support Professor Vanden Berghe’s implication that “plants” (nor primitive organisms such as diatoms) should now be used as indicator species for human safety assessment and, rather, support the current processes in place. In fact two studies that Professor Vanden Berghe cites (Blunder *et al*., 2011 & Arkles *et al*., 2013) did not investigate MMST.

5. Data sheets and analytical reports confirmed the purity of the MMST in aqueous solution with only background potassium and phosphate ions: no other components were present when assessed against stringent analytical criteria.

6. Page 2, line 53 to line 57 of our manuscript [5] explains why pre-menopausal women may be an important target population for Si supplementation. We agree that similar investigations could be undertaken in men and older women, but it is highly unlikely that any potential detrimental effects would be seen in these populations and not in young women. Indeed, we previously reported no difference in the metabolism of MMST or OSA in these different populations [8,16].

7. We disagree with Professor Vanden Berghe’s concerns over our study design. Subjects were used as their own controls against placebo, in a randomised and blinded fashion: this is a standard, robust trial design that allows conclusions to be drawn in comparing the “active” to “placebo” and both against baseline. Given that only the active (MMST), and not placebo, differed from baseline and there were no effects of order (MMST before placebo or placebo before MMST), then clearly the diet over the eight weeks played no significant role. Secondly, previous work, as we have referenced [8,13], characterised the acute urinary excretion of MMST: the ten-hour fast of subjects in our just-published study [5] was well outside of this period. Indeed, that only 10% of the increase in total urinary silicon post supplementation could be accounted for by MMST proves that there was little carry over. Thirdly, the finding of very low urinary levels of MMST in a few volunteers at baseline or after ingesting only placebo indicates *some* normal background environmental exposure. Whilst the source would be of interest, in the context of this study the levels were tiny (21 ± 7 μg/L MMST *versus* 8.5 ± 5 mg/L total Si in the same urine samples) and in no way could therefore contribute to our findings. Fourthly, there was no wash out period (see 'Subjects and methods’ section [5]), and the fact that there was no effect of treatment order on the analytical results justifies this decision.

8. Professor Vanden Berghe refers to a comment on our study that was posted on line by Dr C Exley, who proposes that valid interpretation of our findings would require GFR measures. For three of the outcomes of interest in our publication, namely raised fasting serum Si as a marker of Si stores, serum Si:MMST ratios and the safety of MMST, there is little to be added by such a measure. However, for the fourth outcome, namely Si:MMST ratio in urine following supplementation, additional urinary creatinine measures may have a role-at least to ward off improbable theories of the data being erroneous due to alterations in renal handling. Our long experience with dietary silicon metabolism studies has taught us that inter-individual variation leads to far greater variance than intra-individual urinary Si excretion. Thus we controlled for this variance using a cross over design, such that the same volunteers emptied their bladders at the same time of day on three separate occasions. As noted above we used the robust design of double bind, placebo-controlled intervention: the baseline and placebo intervention yielded no difference in urinary silicon concentrations. As such, for MMST intervention to increase the urinary silicon concentration as an artefact, the supplement would need to cause an unexpected concentration of dissolved urinary solids. Our prior work on MMST absorption and excretion in volunteers has not revealed changes in urinary volume and, as our overall objective was to seek Si:MMST ratios following supplementation, arguments around improvement of GFR would also need to include disproportionate concentration of one form of silicon over the other. The increase in fasting urinary silicon levels mirrored increases in fasting serum silicon levels and we stand fully by the interpretation of these findings.

9. It is not possible that tissue uptake of MMST explains our data. Ingested MMST could not increase total urinary silicon by the amounts shown AND simultaneously be partitioned into tissue. The point we made, that appears to have been missed, is as follows: of the measured increase in urinary silicon, only 10% exists in the form of MMST (either as the tail of the acute excretion or a slower secondary pathway of excretion). Either way, MMST is clearly being converted to inorganic silicon since no other organic form was detected by proton NMR. At these concentrations inorganic silicon only exists as Si(OH)_4_.

10. Regarding the use of ^29^Si NMR for speciation, Professor Vanden Berghe quotes some idealised, non-aqueous limit of *detection* (LOD) work. Under aqueous conditions in biological samples the limit of *quantitation* is greatly compromised. An entire study with pure ^29^Si-supplement would not only be extremely expensive and time consuming but would still provide samples with insufficient ^29^Si for quantitation by the world’s most sensitive ^29^Si NMR spectrometer^a^. We took a pragmatic and robust approach to the problem and used differential analysis (^1^H-NMR versus ICPOES) to demonstrate, albeit indirectly, bioconversion. Coupled with the published direct metabolism data that we reference we firmly conclude that supplemental MMST undergoes metabolism (demethylation and hydroxylation) and forms Si(OH)_4_*in vivo* in humans. Interestingly, the work of Côté-Beaulieu *et al*., (2009) and Arkles *et al*., (2013) that Professor Vanden Berghe cites, also indicate the *in vivo* bioconversion of methylsilanetriols to silicic acid.

## Conclusion

We fully stand by our statements over the safety and metabolism of MMST in the human setting of our study. We urge that with the spotlight on nanosilica safety, other forms of supplemental silicon now provide similar direct safety data.

## Endnotes

^a^In the 1999 paper referred to by Professor Vanden Berghe (Knight CTG, Kinrade SD. Silicon-29 nuclear magnetic resonance spectroscopy detection limits. *Anal Chem* 1999, 71:265-267), the authors reported *optimal*^29^Si NMR limits of detection (LOD), based on a 2:1 signal-to-noise ratio (S/N) and using the highest-field commercial spectrometer available for ^29^Si NMR, for hexamethyldisiloxane dissolved in chloroform. Using a normal one-pulse sequence with a 17 h total run time (6784 90° pulses with a recycle period of 10 s, attenuated by the addition of Cr(acac)_3_ relaxation agent), they achieved a 2 ppm LOD. They managed to reduce the LOD to 0.3 ppm by using a DEPT-45 pulse sequence which transfers polarization to ^29^Si nuclei from coupled methyl protons and also permits much faster pulse recycling. This technique, however, is not possible for orthosilicic acid, owing to rapid exchange of silanol protons with water. Moreover, the T_1_ relaxation time for dilute orthosilicic acid in blood or urine exceeds 30-40 s (Kinrade SD, Marat K, Knight CTG. Longitudinal ^29^Si nuclear magnetic relaxation in aqueous alkali-metal silicate solutions revisited. *J Phys Chem* 1996, 100:18351-18356). Based on the numbers presented above, the theoretical LOD for orthosilicic acid in a biofluid would be approximately 7 ppm (612 90° pulses with a recycle period of 100 s over 17 h). This would be expected to decrease to 0.4 ppm if the orthosilicic acid was 100% enriched in ^29^Si isotope. In practise, however, the best experimental LOD that the same authors have ever achieved in blood, for example, is just under 1 ppm (manuscript in preparation, Kinrade *et al*). Unfortunately, the above LODs are not sufficient for semi-quantitative NMR. High precision (standard deviation <1%) NMR peak integration requires at least a 250:1 S/N (Holzgrabe U. Quantitative NMR spectroscopy in pharmaceutical applications. *Progr Nucl Magn Reson Spectr* 2010, 57:229-240). Even a minimal level of precision would require at least a 25:1 S/N or a biofluid concentration of 25 ppm ^29^Si.

## Competing interests

The authors’ laboratories have received research funding from the silicon supplement and food industry. For the work presented in the just-published paper [5] the MMST and placebo solutions and costs of ^1^H-NMR analysis were provided by LLR-G5 Ltd (Castlebar, Ireland) and all other research costs were met by the author’s laboratories. The research was designed, executed, analysed and communicated only by the authors. J. J. P. and S. D. K. have consulted to BioMinerals NV (Ch-OSA company) and/or LLR-G5 Ltd (MMST company) and others in the silicon supplement industry. All other authors have no competing interest.
